# Trends in Health Service Use for Canadian Adults With Dementia and Parkinson Disease During the First Wave of the COVID-19 Pandemic

**DOI:** 10.1001/jamahealthforum.2021.4599

**Published:** 2022-01-21

**Authors:** Susan E. Bronskill, Laura C. Maclagan, Colleen J. Maxwell, Andrea Iaboni, R. Liisa Jaakkimainen, Connie Marras, Xuesong Wang, Jun Guan, Daniel A. Harris, Abby Emdin, Aaron Jones, Nadia Sourial, Claire Godard-Sebillotte, Isabelle Vedel, Peter C. Austin, Richard H. Swartz

**Affiliations:** 1 ICES, Toronto, Ontario, Canada; 2 Dalla Lana School of Public Health, University of Toronto, Toronto, Ontario, Canada; 3 Sunnybrook Research Institute, Sunnybrook Health Sciences Centre, Toronto, Ontario, Canada; 4 Institute of Health Policy, Management & Evaluation, University of Toronto, Toronto, Ontario, Canada; 5 Schools of Pharmacy and Public Health Sciences, University of Waterloo, Waterloo, Ontario, Canada; 6 KITE Research Institute, Toronto Rehabilitation Institute, University Health Network, Toronto, Ontario, Canada; 7 Department of Psychiatry, University of Toronto, Toronto, Ontario, Canada; 8 Department of Family and Community Medicine, University of Toronto, Toronto, Ontario, Canada; 9 Edmond J. Safra Program in Parkinson Disease, Toronto Western Hospital, Toronto, Ontario, Canada; 10 Department of Health Research Methods, Evidence, and Impact, McMaster University, Hamilton, Ontario, Canada; 11 Department of Health Management, Evaluation and Policy, School of Public Health, University of Montreal, Montreal, Quebec, Canada; 12 Department of Medicine (Geriatrics), McGill University, Montréal, Québec, Canada; 13 Department of Family Medicine, McGill University, Montréal, Québec, Canada; 14 Department of Medicine (Neurology), University of Toronto, Toronto, Ontario, Canada

## Abstract

**Importance:**

Persons with dementia and Parkinson disease (PD) are vulnerable to disruptions in health care and services.

**Objective:**

To examine changes in health service use among community-dwelling persons with dementia, persons with PD, and older adults without neurodegenerative disease during the first wave of the COVID-19 pandemic.

**Design, Setting, and Participants:**

Repeated cross-sectional analysis using population-based administrative data among community-dwelling persons with dementia, persons with PD, and adults 65 years and older at the start of each week from March 1 through the week of September 20, 2020 (pandemic period), and March 3 through the week of September 22, 2019 (historical period), in Ontario, Canada.

**Exposures:**

COVID-19 pandemic as of March 1, 2020.

**Main Outcomes and Measures:**

Main outcomes were weekly rates of emergency department visits, hospitalizations, nursing home admissions, home care, virtual and in-person physician visits, and all-cause mortality. Poisson regression models were used to calculate weekly rate ratios (RRs) with 95% CIs comparing pandemic weeks with historical levels.

**Results:**

Among those living in the community as of March 1, 2020, persons with dementia (n = 131 466; mean [SD] age, 80.1 [10.1] years) were older than persons with PD (n = 30 606; 73.7 [10.2] years) and older adults (n = 2 363 742; 74.0 [7.1] years). While all services experienced declines, the largest drops occurred in nursing home admissions (RR for dementia: 0.10; 95% CI, 0.07-0.15; RR for PD: 0.03; 95% CI, 0.00-0.21; RR for older adults: 0.11; 95% CI, 0.06-0.18) and emergency department visits (RR for dementia: 0.45; 95% CI, 0.41-0.48; RR for PD: 0.40; 95% CI, 0.34-0.48; RR for older adults: 0.45; 95% CI, 0.44-0.47). After the first wave, most services returned to historical levels except physician visits, which remained elevated (RR for dementia: 1.07; 95% CI, 1.05-1.09; RR for PD: 1.10, 95% CI, 1.06-1.13) and shifted toward virtual visits. Older adults continued to experience lower hospitalizations. All-cause mortality was elevated across cohorts.

**Conclusions and Relevance:**

In this population-based repeated cross-sectional study in Ontario, Canada, those with dementia, those with PD, and older adults sought hospital care far less than usual, were not admitted to nursing homes, and experienced excess mortality during the first wave of the pandemic. Most services returned to historical levels, but virtual physician visits remained a feature of care. While issues of equity and quality of care are still emerging among persons with neurodegenerative diseases, policies to support virtual care are necessary.

## Key Points

**Question:**

Was the COVID-19 pandemic associated with changes in health service use and mortality among community-dwelling persons with dementia and Parkinson disease compared with older adults?

**Findings:**

In this population-based repeated cross-sectional analysis, large declines in hospital use and nursing home admission were experienced across all cohorts. After the first wave, most services returned to historical levels, with physician visits elevated and mostly virtual, nursing home admissions reduced, and excess all-cause mortality.

**Meaning:**

The pandemic was associated with meaningful health service disruptions for persons with dementia and Parkinson disease, highlighting that continued support for virtual care is needed to ensure optimal health outcomes.

## Teaser

This cross-sectional analysis examines changes in health service use among community-dwelling persons with dementia, persons with Parkinson disease, and older adults without neurodegenerative disease during the first wave of the COVID-19 pandemic.

## Introduction

The COVID-19 pandemic continues to strain health systems internationally as deaths rise and countries struggle to care for infected individuals with limited resources. The pandemic also disrupted provision of non–COVID-19–related health care services.^1,2,3^ Importantly, many physicians and other health care professionals shifted to virtual care owing to safety concerns and transmission risks.^4,5,6^ In the pandemic’s early stages, hospitals discharged individuals to prepare for patients with COVID-19,^7^ surgical procedures were canceled,^8^ and individuals were hesitant to visit emergency departments.^9,10^ In the home care setting, clients canceled in-home visits owing to transmission fears, and clinicians faced barriers to care provision, including a lack of access to personal protective equipment and safe transportation.^11,12,13^ The COVID-19 pandemic has been particularly devastating in the nursing home setting, with high mortality rates^14,15,16^ prior to vaccine distribution.

Continuity of care and sustained access to health services, including primary care, specialists, and home care, are important for persons living in community settings with neurodegenerative diseases, such as Alzheimer and related dementias (dementia)^17,18,19^ and Parkinson disease (PD).^20^ In Canada, the COVID-19 pandemic took a large toll on older adults, and neurodegenerative disease was a common comorbidity among those who died.^21^ Health service disruptions and communication challenges introduced by the pandemic have the potential to create gaps in care that could be challenging for persons with dementia,^19,22^ persons with PD,^23,24,25^ and their caregivers.^26^ Most studies to date have examined health service use for single services,^6,7,8^ and to our knowledge, there has not yet been a comprehensive assessment quantifying the magnitude of service disruptions among individuals with dementia and PD during the first wave of the pandemic across a spectrum of services, including in-person and virtual services.

In this context, we sought to compare patterns of health service use and all-cause mortality among community-dwelling persons with dementia, persons with PD, and older adults without neurodegenerative disease to prepandemic levels. This cross-sectoral approach highlights the codependence of health services and may guide health systems in anticipating how to support vulnerable populations through future pandemic waves and other emergencies.

## Methods

### Study Design and Setting

We conducted a population-based repeated cross-sectional study of health service use among persons with dementia and PD and a comparison cohort of older adults without neurodegenerative disease using health administrative data from Ontario, Canada. We compared changes in weekly rates of health service use during the first 6 months of the pandemic to the same period in 2019, by type of service. Ontario is Canada’s most populous province with a population of nearly 14.6 million residents. Nearly all Ontario residents receive universal health insurance coverage for medically necessary services, including physician visits, hospitalizations, emergency department visits, and home care services.

We used the Ontario Registered Persons Database, a registry of all Ontarians eligible for provincial health insurance, to identify persons alive and eligible for health services at the start of each week and to obtain demographic information, including age, sex, and death date, if applicable. The Canadian Institute for Health Information Discharge Abstract Database and the National Ambulatory Care Reporting System includes hospital records and were used to identify hospitalizations and emergency department visits, respectively. The Ontario Health Insurance Plan (OHIP) Claims Database includes billings for health care professionals and was used to identify physician visits. The Health Shared Services Ontario Home Care Database was used to identify publicly funded home care services. Residence in a nursing home was determined using the Continuing Care Reporting System Long-Term Care Database, a registry of all individuals residing in nursing homes in Ontario, combined with information from OHIP claims and Ontario Drug Benefit Program drug dispensation claims.^27^ All data sets were linked using unique encoded identifiers and analyzed at ICES. ICES is an independent, nonprofit research institute whose legal status under Ontario’s health information privacy law allows it to collect and analyze health care and demographic data, without consent, for health system evaluation and improvement. The use of data in this project is authorized under section 45 of Ontario’s Personal Health Information Protection Act and does not require review by a research ethics board. All methods and outcomes are reported according to the best practices described in the Reporting of Studies Conducted Using Observational Routinely Collected Health Data (RECORD) statement, an extension of Strengthening the Reporting of Observational Studies in Epidemiology (STROBE) reporting guidelines.^28^

### Study Population

We designated a pandemic period (from March 1, 2020, through the week of September 20, 2020) and a historical comparison period (from March 3, 2019, through the week of September 22, 2019). While the first case of COVID-19 was confirmed in Ontario on January 25, 2020, widespread community transmission was not evident until March 2020. Individuals who were aged 40 years and older and living with dementia and/or PD at the start of each week were identified using validated health administrative data algorithms^29,30^ (eTable 1 in the Supplement). A comparison group of older adults aged 65 years and older (without documented dementia, PD, or amyotrophic lateral sclerosis) was selected to examine changes in health system use during the pandemic among older adults who were not living with neurodegenerative diseases. We excluded individuals who resided in nursing homes at the start of each week.

We described age, sex, rurality of residence, neighborhood income quintile, disease duration, and number of chronic conditions for each cohort during the first week of each period. Rurality was determined by the Rurality Index of Ontario, an index based on population factors and distance to referral centers, ranging from 0 to 100, with values of 40 or higher considered rural.^31^ Neighborhood income quintile was captured by linking to Statistics Canada census data using postal codes. Disease duration was calculated as time from case ascertainment with administrative data (ie, date of the first health system encounter meeting criteria for algorithm) to the first week in the pandemic and historical periods. Prevalence of 18 chronic conditions and a summary comorbidity count were estimated using commonly used (and where available, validated) administrative data algorithms.^32^

### Outcomes

The primary outcomes were weekly rates of all-cause health service use and mortality. Health services included emergency department visits (not resulting in a hospitalization), hospitalizations (including admissions, discharges, and delayed discharges), physician visits (including family physicians, neurologists, geriatricians, psychiatrists, and other specialists), home care visits, and nursing home admissions. Discharges with a delay indicate that a patient did not require the intensity of resources/services provided in the hospital but was unable to be discharged for other reasons (eg, lack of available community or nursing home care).^33^ Discharges with a delay were identified from relevant variables in hospital discharge records. Physician visits could be virtual or in-person. Virtual physician visits were defined using fee codes indicating a telephone or video visit in the OHIP database. In-person physician visits were defined as those provided in office and home locations. Home care services include all publicly funded homemaking, transportation, personal care, nursing care, end-of-life care, physiotherapy, and occupational and speech-language therapy.

### Statistical Analysis

Patient demographic characteristics and chronic conditions were summarized at the start of the pandemic and historical periods. Rates of health service use and mortality per 100 persons were calculated for each week of the pandemic period (and equivalent weeks in the historical period), with a focus on the earliest and latest weeks and the weeks with the minimum and maximum rates during the pandemic. Poisson regression models with an offset term for the number of individuals at risk each week and a single indicator variable for year (pandemic vs historical period) were used to calculate weekly rate ratios (RRs) and 95% CIs. All *P* values were 2-tailed, and values .05 or less were considered statistically significant. These models did not include adjustment for any additional covariates because the aim of the study was descriptive^34^; however, mortality analyses were further stratified by age. Cumulative change in the number of outcomes was calculated as the difference in total events between the pandemic and historical periods. Analyses were conducted using SAS, version 9.4 (SAS Institute).

## Results

We identified 131 466 persons with dementia (mean [SD] age, 80.1 [10.1] years), 30 606 persons with PD (mean [SD] age, 73.7 [10.2] years), and 2 363 742 older adults (74.0 [7.1] years) living in the community on March 1, 2020, and 133 142 persons with dementia (mean [SD] age, 80.1 [10.1] years), 30 052 persons with PD (mean [SD] age, 73.6 [10.3] years), and 2 245 915 older adults (mean [SD] age, 73.8 [7.1] years) on March 3, 2019 (Table 1). Some individuals had both conditions (10 873 in 2019 and 9957 in 2020), reflecting neurodegenerative multimorbidity. Within each cohort, distributions of age, sex, and chronic conditions were similar between 2019 and 2020. As expected, persons with dementia were older and had higher comorbidity, and those with PD were more likely to be male. Older adults were more likely to live in a rural location. At the start of the pandemic, the mean (SD) disease duration was 4.5 (4.3) years in those with dementia and 6.3 (5.6) years in those with PD.

**Table 1.** Baseline Characteristics of Community-Dwelling Persons With Dementia, Persons With Parkinson Disease, and Older Adults During the Historical and COVID-19 Pandemic Periods in Ontario, Canada^a^

Characteristic	Dementia		Parkinson disease		Older adults^b^	
	2019	2020	2019	2020	2019	2020
No.	133 142	131 466	30 052	30 606	2 245 915	2 363 742
Age, y						
Mean (SD)	80.1 (10.1)	80.1 (10.1)	73.6 (10.3)	73.7 (10.2)	73.8 (7.1)	74.0 (7.1)
Median (IQR)	82 (74-87)	82 (74-87)	74 (67-81)	74 (67-81)	72 (68-78)	72 (68-78)
Age groups, No. (%)						
40-64 y	10 938 (8.2)	10 717 (8.2)	5564 (18.5)	5545 (18.1)	NA	NA
65-74 y	22 801 (17.1)	22 772 (17.3)	9689 (32.2)	9849 (32.2)	1 370 378 (61.0)	1 430 108 (60.5)
75-84 y	49 609 (37.3)	48 065 (37.3)	10 455 (34.8)	10 822 (35.4)	656 921 (29.2)	695 647 (29.4)
≥85 y	49 794 (37.4)	48 912 (37.2)	4344 (14.5)	4390 (14.3)	218 616 (9.7)	237 987 (10.1)
Sex						
Female	76 428 (57.4)	75 538 (57.5)	12 297 (40.9)	12 593 (41.1)	1 207 166 (53.7)	1 271 551(53.8)
Male	56 714 (42.6)	55 928 (42.5)	17 755 (59.1)	18 013 (58.9)	1 038 749 (46.3)	1 092 191(46.2)
Rural	12 049 (9.3)	12 088 (9.2)	3148 (10.5)	3197 (10.4)	287 354 (12.8)	302 649 (12.8)
Income quintile, No. (%)						
1 (Lowest)	30 858 (23.2)	29 937 (22.8)	5902 (19.6)	5920 (19.3)	433 922 (19.3)	452 636 (19.1)
2	28 905 (21.7)	28 482 (21.7)	6045 (20.1)	6185 (20.2)	463 900 (20.7)	485 801 (20.6)
3	25 714 (19.3)	25 363 (19.3)	5976 (19.9)	6030 (19.7)	450 174 (20.0)	474 224 (20.1)
4	23 329 (17.5)	23 246 (17.7)	5707 (19.0)	5828 (19.0)	430 892 (19.2)	456 427 (19.3)
5 (Highest)	23 603 (17.7)	23 714 (18.0)	6319 (21.0)	6539 (21.4)	461 369 (20.5)	488 684 (20.7)
Disease duration, y, mean (SD)	4.2 (4.2)	4.5 (4.3)	6.1 (5.5)	6.3 (5.6)	NA	NA
No. of chronic conditions^c^						
Mean (SD)	5.0 (2.0)	4.9 (2.0)	3.5 (2.0)	3.5 (2.0)	2.9 (1.8)	2.9 (1.8)
Counts						
0-1	2949 (2.2)	3373 (2.6)	4896 (16.3)	5192 (17.0)	517 579 (23.0)	558 203 (23.6)
2	9806 (7.4)	9997 (7.6)	5339 (17.8)	5545 (18.1)	504 178 (22.4)	533 997 (22.6)
3	19 635 (14.7)	19 804 (15.1)	5888 (19.6)	6064 (19.8)	484 748 (21.6)	508 528 (21.5)
4	26 039 (19.6)	25 902 (19.7)	5294 (17.6)	5245 (17.1)	345 135 (15.4)	358 310 (15.2)
≥5	74 713 (56.1)	72 390 (55.1)	8635 (28.7)	8560 (28.0)	394 275 (17.6)	404 704 (17.1)

Abbreviation: NA, not applicable.

^a^ 2019 refers to the historical period as of March 3, 2019; 2020, the COVID-19 pandemic period as of March 1, 2020.

^b^ Older adults are defined as persons aged 65 years and older without a diagnosis of neurodegenerative diseases.

^c^ Chronic conditions include acute myocardial infarction (AMI); osteoarthritis; other arthritis; rheumatoid arthritis; asthma; cancer; cardiac arrhythmia; heart failure; chronic obstructive pulmonary disease; coronary syndrome (excluding AMI); dementia; diabetes; hypertension; mood, anxiety, depression, and other nonpsychotic disorders; other mental illnesses; osteoporosis; kidney failure; stroke (excluding transient ischemic attack).

### Magnitude of Initial Decline Across Services

**Figure 1.**
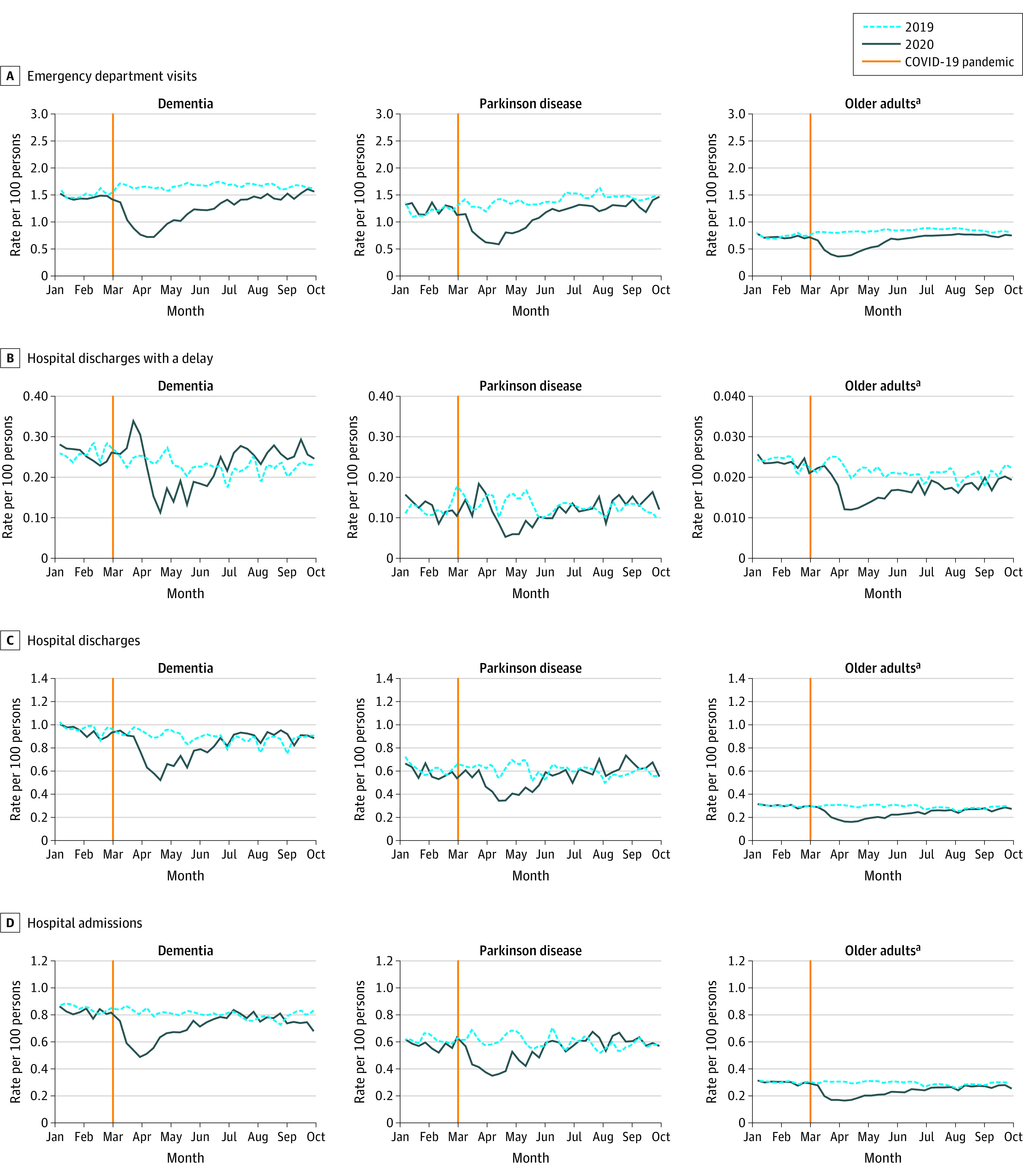
Rates of Hospital Use by Community-Dwelling Persons With Dementia, Persons With Parkinson Disease, and Older Adults, 2019-2020 ^a^Older adults are defined as persons aged 65 years and older without a diagnosis of neurodegenerative disease.

**Figure 2.**
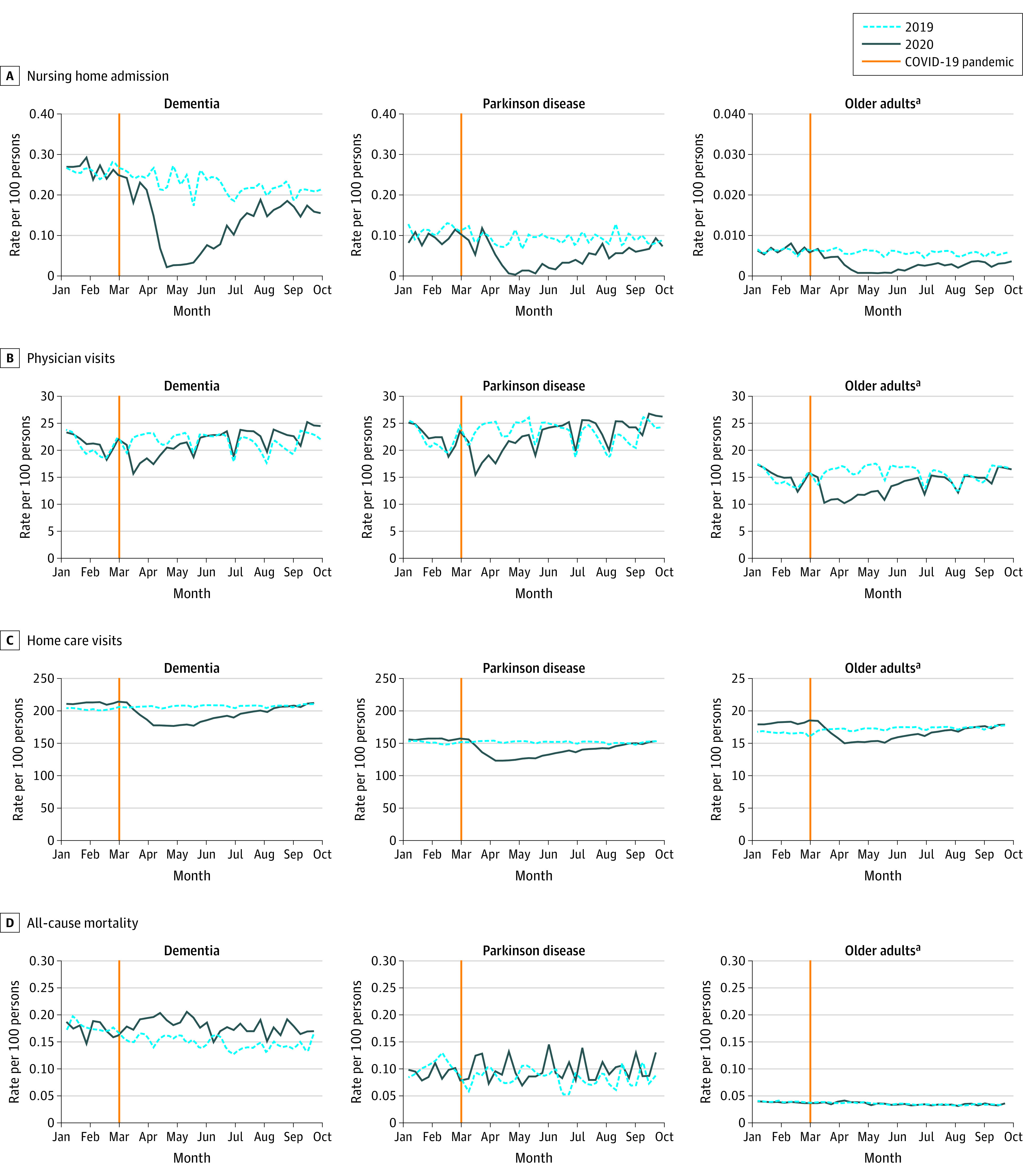
Rates of Health Service Use and Mortality by Community-Dwelling Persons With Dementia, Persons With Parkinson Disease, and Older Adults, 2019-2020 ^a^Older adults are defined as persons aged 65 years and older without a diagnosis of neurodegenerative disease.

Across cohorts and services, there were large, immediate decreases in health service use (Figure 1 and Figure 2). The date of the lowest weekly rate varied by cohort and service (eTable 2 in the Supplement) but typically occurred in late March or early April 2020. An exception to this was hospital discharges with a delay for persons with dementia and PD, which rose before declining. Nursing home admissions had the largest relative decline and all but ceased through May and June 2020 (RR for dementia: 0.10; 95% CI, 0.07-0.15; RR for PD: 0.03; 95% CI, 0.00-0.21; RR for older adults: 0.11; 95% CI, 0.06-0.18) (Table 2). Emergency department visits had large declines (RR for dementia: 0.45; 95% CI, 0.41-0.48; RR for PD: 0.40; 95% CI, 0.34-0.48; RR for older adults: 0.45; 95% CI, 0.44-0.47) and were similar across cohorts. Total physician visits experienced smaller declines and varied by cohort (RR for dementia: 0.70; 95% CI, 0.68-0.71; RR for PD: 0.66; 95% CI, 0.64-0.69; RR for older adults: 0.59; 95% CI, 0.59-0.59). Physician visit patterns differed by specialty, with family physicians experiencing a smaller decline than specialists. Home care visits experienced the smallest declines (RR for dementia: 0.85; 95% CI, 0.84-0.85; RR for PD: 0.80, 95% CI, 0.79-0.81; RR for older adults: 0.87; 95% CI, 0.86-0.87).

**Table 2.** Rate Ratios of Health Service Use by Community-Dwelling Persons With Dementia, Persons With Parkinson Disease, and Older Adults Comparing the COVID-19 Pandemic Period With the Historical Period in Ontario, Canada

Health service/outcome	Rate ratios (95% CI)		Cumulative change in No. of outcomes, No.^c^
	At lowest rate in 2020^a^	At latest week^b^	
**Dementia**			
Emergency department visits	0.45 (0.41-0.48)	0.98 (0.93-1.04)	−18 380
Hospital			
Admissions	0.61 (0.55-0.67)	0.94 (0.86-1.03)	−4342
Discharges	0.58 (0.53-0.64)	1.00 (0.93-1.09)	−3755
Discharges with a delay	0.46 (0.38-0.56)	1.11 (0.95-1.30)	−216
Visits			
Total physician	0.70 (0.68-0.71)	1.07 (1.05-1.09)	−33 848
Family physician	0.78 (0.76-0.80)	1.10 (1.08-1.12)	17 043
Total specialist	0.56 (0.54-0.57)	1.03 (1.00-1.05)	−50 892
Neurologist	0.54 (0.48-0.62)	1.31 (1.18-1.45)	−1064
Geriatrician/psychiatrist	0.60 (0.56-0.65)	1.11 (1.05-1.18)	−1044
Other specialist^d^	0.55 (0.53-0.57)	0.99 (0.96-1.02)	−48 785
Home care	0.85 (0.84-0.85)	1.01 (1.00-1.01)	−721 240
Nursing home admissions	0.10 (0.07-0.15)	0.76 (0.64-0.91)	−3954
Mortality	1.40 (1.16-1.68)^a^	1.04 (0.86-1.25)	1092
**Parkinson disease**			
Emergency department visits	0.40 (0.34-0.48)	0.96 (0.84-1.10)	−2887
Hospital			
Admissions	0.60 (0.47-0.76)	1.02 (0.83-1.26)	−535
Discharges	0.66 (0.51-0.84)	1.23 (1.00-1.51)	−542
Discharges with a delay	0.36 (0.20-0.64)	1.47 (0.95-2.28)	−116
Visits			
Total physician	0.66 (0.64-0.69)	1.10 (1.06-1.13)	−7283
Family physician	0.75 (0.72-0.79)	1.08 (1.04-1.13)	3377
Total specialist	0.55 (0.52-0.59)	1.12 (1.07-1.17)	−10 661
Neurologist	0.52 (0.46-0.58)	1.52 (1.39-1.67)	215
Geriatrician/psychiatrist	0.63 (0.53-0.75)	1.21 (1.04-1.40)	331
Other specialist^d^	0.55 (0.52-0.60)	0.98 (0.92-1.04)	−11 207
Home care	0.80 (0.79-0.81)	1.00 (0.99-1.01)	−118 851
Nursing home admissions	0.03 (0.00-0.21)	1.14 (0.67-1.96)	−399
Mortality	1.61 (1.00-2.60)^a^	1.44 (0.88-2.36)	150
**Older adults** ^e^			
Emergency department visits	0.45 (0.44-0.47)	0.91 (0.89-0.93)	−108 900
Hospital			
Admissions	0.54 (0.52-0.56)	0.94 (0.90-0.97)	−31 075
Discharges	0.54 (0.52-0.56)	0.96 (0.92-0.99)	−30 034
Discharges with a delay	0.61 (0.53-0.71)	0.87 (0.77-0.98)	−2111
Visits			
Total physician	0.59 (0.59-0.59)	0.99 (0.98-0.99)	−1 108 852
Family physician	0.63 (0.62-0.63)	1.00 (1.00-1.01)	−424 470
Total specialist	0.54 (0.53-0.54)	0.96 (0.96-0.97)	−684 390
Neurologist	0.61 (0.57-0.65)	1.15 (1.09-1.21)	−4679
Geriatrician/psychiatrist	0.72 (0.69-0.75)	1.10 (1.06-1.14)	13 717
Other specialist^d^	0.53 (0.52-0.53)	0.96 (0.95-0.96)	−693 430
Home care	0.87 (0.86-0.87)	1.01 (1.00-1.01)	68 776
Nursing home admissions	0.11 (0.06-0.18)	0.55 (0.41-0.72)	−2001
Mortality	1.12 (1.02-1.23)^a^	1.10 (1.00-1.22)	1481

^a^ For all-cause mortality, the pandemic period rates increased (rather than decreased); therefore, rate ratios for the highest week are presented.

^b^ The latest weekly rate is as of September 20, 2020, and September 22, 2019, for all services.

^c^ The cumulative change in the number of outcomes was calculated comparing the number of events within the pandemic period (March 1 to September 20, 2020) and historical period (March 3 and September 22, 2019). Negative values indicate a decreased number of events in the 2020 COVID-19 pandemic period.

^d^ Other specialist includes specialists other than neurologists, geriatricians, and psychiatrists.

^e^ Older adults include persons aged 65 years and older without a diagnosis of neurodegenerative diseases.

### Magnitude of Decline by the End of the First Wave Across Services

By the end of the study period, most services had rebounded to historical levels, except for nursing home admissions (Figures 1 and 2, Table 2). Weekly mortality rates showed increases in all cohorts, with the largest relative increase among persons with dementia and PD and a smaller increase in older adults (at the highest week, RR for dementia: 1.40; 95% CI, 1.16-1.68; RR for PD: 1.61; 95% CI, 1.00-2.60; RR for older adults: 1.12; 95% CI, 1.02-1.23). eTable 3 in the Supplement shows these findings by age strata, which were statistically significant in persons with dementia, and the largest RR was for those younger than 75 years (2.57; 95% CI, 1.41-4.67). By the end of the study period, weekly RRs for mortality in persons with dementia and persons with PD were no longer significant. There were also large changes in the magnitude of service use (eg, −18 380 emergency department visits for persons with dementia) (Table 2). We observed excess deaths in all cohorts over the study period (+1092 dementia; +150 PD; +1481 older adults).

### Shift to Virtual Physician Visits

**Figure 3.**
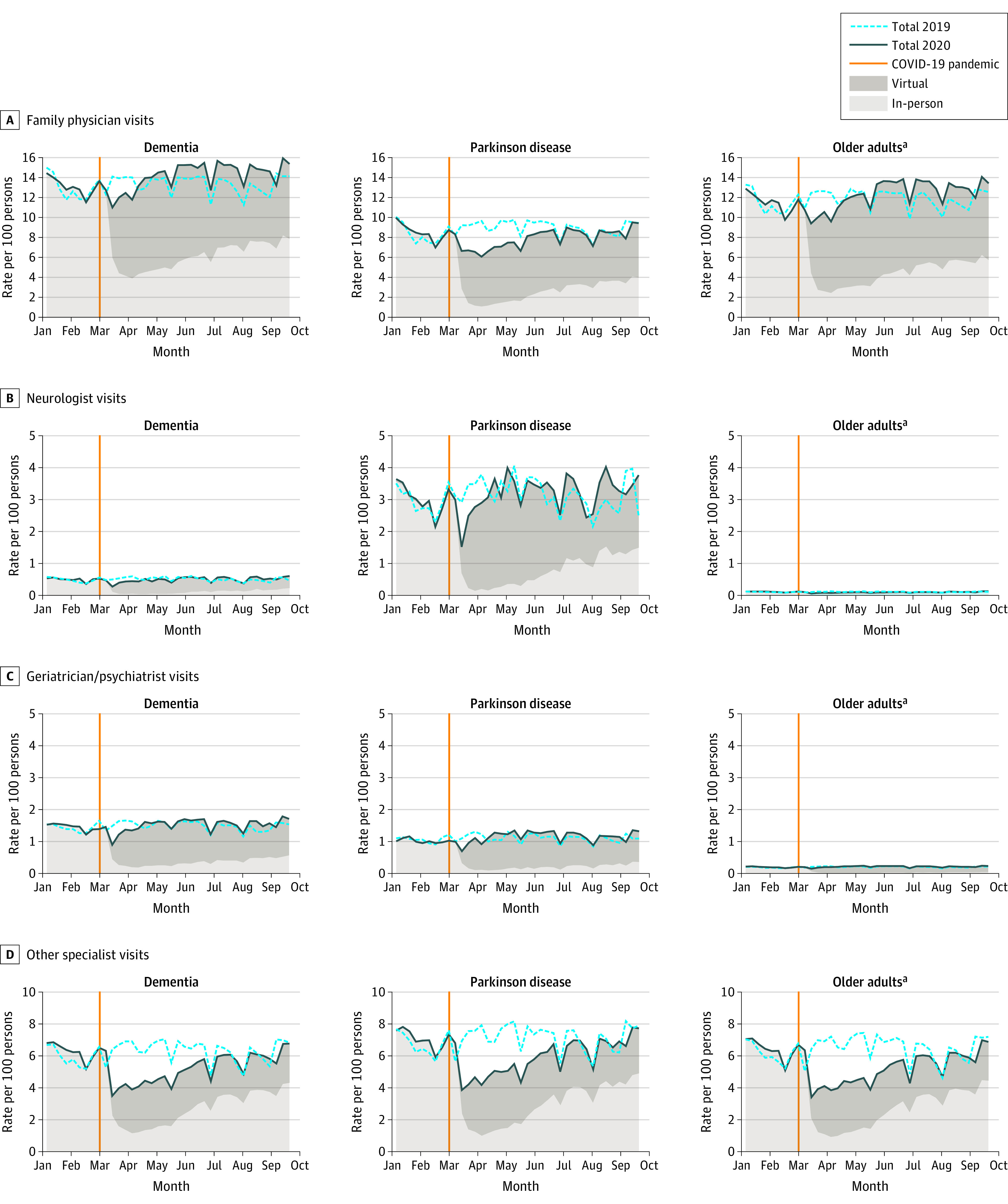
Rates of Physician Visits by Community-Dwelling Persons With Dementia, Persons With Parkinson Disease, and Older Adults, 2019-2020 ^a^Older adults are defined as persons aged 65 years and older without a diagnosis of neurodegenerative disease.

At the end of the study period, physician visits remained elevated across all cohorts, particularly family physician visits for those with dementia (RR, 1.10; 95% CI, 1.08-1.12) and PD (RR, 1.08; 95% CI, 1.04-1.13). There was a rapid shift toward virtual visits across all physician specialties and a slow reintroduction of in-person visits (Figure 3). The return to in-person visits varied by cohort and specialty.

## Discussion

To our knowledge, these findings are the first population-level assessment quantifying the magnitude of changes in health service use in persons with dementia, persons with PD, and older adults without neurodegenerative disease during the first wave of the COVID-19 pandemic across a spectrum of services. While all services experienced immediate declines, nursing home admissions all but stopped (and remained low), and emergency department visits experienced large declines. After the first wave, most services returned to historical levels; however, physician visits exceeded these levels, and virtual visits remained a feature of care. Changes in service patterns were similar across cohorts. Exceptions included initial increases in delayed hospital discharges for those with dementia and PD, and hospitalizations remaining below historical levels at the end of the study period in older adults. Findings from the present study demonstrate that, even in a universal health care system such as Canada’s, health sectors experienced the pandemic differently, and reactions were siloed.

Excess mortality occurred for all cohorts over the study period, but the largest relative effect occurred in persons with dementia and PD. However, these effects had overlapping CIs, with the significant increase also observed in older adults. Additional research is needed to disentangle deaths directly attributable to COVID-19 vs those that may have occurred owing to other causes. We note many excess deaths in those with dementia, particularly those younger than 75 years where prepandemic mortality rates were low. In Ontario, many community-dwelling older adults with dementia live in congregate living settings, such as retirement homes, which provide specialized accommodation and supports.^35^ COVID-19 had devastating outcomes in both the nursing home^15^ and retirement home^36^ sectors globally owing to challenges in infection control, issues with staffing and staff transmission,^37,38^ and limitations placed on family and friends entering facilities.^39^ While the present study’s mortality findings in those with dementia likely highlight the outcomes of COVID-19 in the retirement home setting, individuals with dementia may have also had a higher mortality risk during the pandemic associated with decreased access to community support and hospital care.

The trends we report are consistent with patterns for other health conditions^3^ and services^6,7,40^ across jurisdictions. In Ontario, system changes were precipitated by the first case announced on January 25, 2020, by the introduction of physician billing codes to facilitate virtual care effective March 14, 2020, and a directive from the Ontario government to halt elective surgical procedures and other nonessential health services.^41^ For most services, we did not see differences in patterns between persons with dementia, persons with PD, and the older adult population in Ontario. This suggests that differential barriers in access to services were not present at the population level.^42^ However, we cannot assess variation in quality or timeliness of service provided, which may have been poorer for those with dementia and PD.

In-depth studies within dementia and PD populations should examine the characteristics of those who sought care, reasons for their visits, and whether changes in later care needs were observed. This will help assess the quality of care provided as well as outcomes of pandemic-related reductions in supportive programs and family/friend caregiving.^22^ Given the significant drop in nursing home admissions and relative stability in home care services for individuals with dementia and PD, it is possible many caregivers tried to manage care at home with little to no help. This could have devastating implications on health and well-being^26^ over the long term. Home care visits should be prioritized in policy decision-making during future pandemic waves, especially during periods in which nursing home admission is not possible. Coordination across sectors is required to plan for the care of persons with neurodegenerative diseases.

Hospitalization patterns reflect both directives associated with preparing for the pandemic^41^ and the outcomes of COVID-19 on Ontario nursing homes and nursing home bed availability.^43^ Early on, hospitals were asked to accommodate patients with COVID-19 and did so by discharging individuals who no longer required hospital services. This is reflected in the initial rise in hospital discharges with a delay. A large proportion of these patients are individuals with dementia^33^ who could not return home and were awaiting care in an alternate setting, frequently in retirement and nursing homes. Through the summer, hospital admissions and discharges returned to historical levels in those with dementia and PD; however, discharges with a delay eventually exceed historical levels in those with dementia. We suspect this is owing to interrelated challenges: (1) nursing homes badly affected by the first wave were required to reduce overcrowding, which reduced capacity; (2) other nursing homes chose to close units to create designated isolation units; (3) there was reluctance among nursing homes to admit people with dementia because of directives to quarantine newly admitted patients. Hospital admissions remained lower in older adults by the end of the study period, likely reflecting delays as clinical services were restarting and hesitancy to seek hospital services. Appropriate messaging surrounding the use of hospital services should be provided so that persons with dementia and PD do not delay seeking necessary care.

The immediate, broad shift to virtual care among physicians has been championed as a success of pandemic planning in Ontario.^5,6^ Findings of the present study suggest that physician visits returned to historical levels faster than other services; this was particularly noticeable in specialist care. Among all specialties, virtual visits represented a large proportion of visits at the end of the study period. This dramatic and rapid shift suggests willingness among both physicians and patients to engage in virtual care. Policy changes implemented in Ontario and other jurisdictions to allow for reimbursement of virtual care services were initially a temporary measure during the pandemic.^44^ These changes will need continued support, particularly for vulnerable populations. The association between virtual health care delivery (vs in-person delivery) and patient outcomes should also be evaluated to clarify the relative benefits and risks, including whether virtual care may help to avoid potentially unnecessary emergency department visits and costly health system use. While virtual primary care may be suitable for individuals with less complex care needs, its appropriateness and role in supporting care continuity for persons with dementia^45,46^ and PD^47^ requires monitoring.

### Limitations

A limitation to our study is the administrative data definitions of dementia and PD. Although validated in a primary care sample,^29,30^ they are not clinical diagnoses, and there is the potential for misclassification. In addition, we lacked information on the reasons for, appropriateness of, and/or quality of care provided to those with dementia, those with PD, and older adults during the pandemic. This is a common limitation with administrative data,^48^ and investigations require additional data sources. Finally, we are currently not able to distinguish individuals living in single-family dwellings from those living in non-nursing home congregate care (eg, retirement homes).

## Conclusions

In this repeated cross-sectional study, the early wave of the COVID-19 pandemic was associated with meaningful and immediate changes in the use of health care services in Ontario for persons with dementia, persons with PD, and other older adults without neurodegenerative disease. While service levels gradually returned to historical levels after the first wave, changes in health service use during subsequent waves of the pandemic continue to emerge. The initial shift to virtual physician care requires support to ensure ongoing equitable and effective care to prevent persons with dementia and PD from experiencing unintended consequences owing to lack of in-person visits.

## Notes

Supplement. **eTable 1.** Administrative data algorithms for neurodegenerative diseases **eTable 2.** Rates of health service use for key weeks by community-dwelling persons with dementia, persons with Parkinson disease and older adults during the historical and pandemic periods in Ontario, Canada **eTable 3.** Rate ratios for mortality in community-dwelling persons with dementia, persons with Parkinson disease and older adults comparing the COVID-19 pandemic period with the historical period in Ontario, Canada, by age group
