# Supplementary material for: Trends in Health Service Use for Canadian Adults With Dementia and Parkinson Disease During the First Wave of the COVID-19 Pandemic
Source: JAMA Health Forum. 2022 Jan 21;3(1):e214599. doi: 10.1001/jamahealthforum.2021.4599 (PMC8903126; doi:10.1001/jamahealthforum.2021.4599)
Supplement: Supplement. — eTable 1. Administrative data algorithms for neurodegenerative diseases eTable 2. Rates of health service use for key weeks by community-dwelling persons with dementia, persons with Parkinson disease and older adults during the historical and pandemic periods in Ontario, Canada eTable 3. Rate ratios for mortality in community-dwelling persons with dementia, persons with Parkinson disease and older adults comparing the COVID-19 pandemic period with the historical period in Ontario, Canada, by age group [file jamahealthforum-e214599-s001.pdf]

Supplemental Online Content

Bronskill SE, Maclagan LC, Maxwell CJ, et al. Trends in health service use for Canadian adults with dementia and Parkinson disease during the first wave of the COVID-19 pandemic. *JAMA Health Forum*. 2022;3(1):e214599. doi:10.1001/jamahealthforum.2021.4599

eTable 1. Administrative data algorithms for neurodegenerative diseases

eTable 2. Rates of health service use for key weeks by community-dwelling persons with dementia, persons with Parkinson disease and older adults during the historical and pandemic periods in Ontario, Canada

eTable 3. Rate ratios for mortality in community-dwelling persons with dementia, persons with Parkinson disease and older adults comparing the COVID-19 pandemic period with the historical period in Ontario, Canada, by age group

This supplemental material has been provided by the authors to give readers additional information about their work.

© 2022 Bronskill SE et al. *JAMA Health Forum.*

eTable 1. Administrative data algorithms for neurodegenerative diseases

	Algorithm	Validated
**Alzheimer's and related dementias**[1]	1 hospitalization or same-day surgery for dementia *and/or* 3 physician visits for dementia within 2 years (each at least 30 days apart) *and/or* filled prescription for a cholinesterase inhibitor	Yes, Se=79.3%, Sp=99.1%, PPV=80.4%, NPV=99.0%
**Parkinson disease**[2]	3 physician visits for Parkinson's disease within 2 years (each at least 30 days apart)	Yes, Se=72.3%, Sp=100.0%, PPV=82.1%, NPV=99.9%
**Amyotrophic lateral sclerosis (ALS)**	Filled prescription for Riluzole with an electromyography test prior to first prescription	No

Se= sensitivity; Sp=specificity; PPV= positive predictive value; NPV=negative predictive value

References

1. Jaakkimainen RL, Bronskill SE, Tierney MC, et al. Identification of physician-diagnosed Alzheimer's disease and related dementias in population-based administrative data: a validation study using family physicians' electronic medical records. *Journal of Alzheimer's Disease.* 2016;54(1):337-349.
2. Maclagan LC, Visanji NP, Cheng Y, et al. Identifying drugs with disease-modifying potential in Parkinson's disease using artificial intelligence and pharmacoepidemiology. *Pharmacoepidemiology and drug safety.* 2020;29(8):864-872.

© 2022 Bronskill SE et al. *JAMA Health Forum.*

eTable 2. Rates of health service use for key weeks by community-dwelling persons with dementia, persons with Parkinson disease and older adults[a] during the historical and pandemic periods[b] in Ontario, Canada

Rate per 100 persons per week	Dementia 2019	Dementia 2020	Parkinson Disease 2019	Parkinson Disease 2020	Older Adults 2019	Older Adults 2020
**Emergency department visits**						
Starting rate[c]	1.52	1.42	1.30	1.13	0.75	0.72
Week of minimum rate[d]		12APR2020		12APR2020		29MAR2020
Minimum rate	1.62	0.72	1.45	0.59	0.79	0.36
Week of maximum rate[d]		20SEP2020		30AUG2020		02AUG2020
Maximum rate	1.64	1.61	1.44	1.42	0.88	0.78
Latest rate[e]	1.64	1.61	1.46	1.40	0.84	0.76
**Hospital admissions**						
Starting rate[c]	0.85	0.82	0.63	0.63	0.31	0.29
Week of minimum rate[d]		29MAR2020		05APR2020		05APR2020
Minimum rate	0.80	0.49	0.58	0.35	0.31	0.16
Week of maximum rate[d]		05JUL2020		19JUL2020		01MAR2020
Maximum rate	0.82	0.84	0.57	0.67	0.31	0.29
Latest rate[e]	0.79	0.74	0.58	0.59	0.30	0.28
**Hospital discharges**						
Starting rate[c]	0.96	0.93	0.66	0.54	0.30	0.30
Week of minimum rate[d]		19APR2020		12APR2020		12APR2020
Minimum rate	0.90	0.52	0.52	0.34	0.30	0.16
Week of maximum rate[d]		23AUG2020		23AUG2020		01MAR2020
Maximum rate	0.87	0.95	0.56	0.73	0.30	0.30
Latest rate[e]	0.90	0.91	0.55	0.67	0.30	0.28
**Hospital discharges with a delay**						
Starting rate[c]	0.27	0.26	0.18	0.10	0.02	0.02
Week of minimum rate[d]		19APR2020		19APR2020		12APR2020
Minimum rate	0.24	0.11	0.15	0.05	0.02	0.01
Week of maximum rate[d]		22MAR2020		22MAR20200		15MAR2020
Maximum rate	0.25	0.34	0.12	0.18	0.02	0.02
Latest rate[e]	0.23	0.26	0.11	0.16	0.02	0.02
**Total physician visits**						
Starting rate[c]	22.54	22.05	24.65	23.48	16.20	15.75
Week of minimum rate[d]		15MAR2020		15MAR2020		05APR2020
Minimum rate	22.47	15.65	23.4	15.46	17.2	10.19
Week of maximum rate[d]		13SEP2020		13SEP2020		13SEP2020
Maximum rate	23.25	25.01	25.52	26.61	16.98	16.85

© 2022 Bronskill SE et al. *JAMA Health Forum.*

Latest rate[e]	22.97	24.39	24.01	26.19	16.98	16.62
**Total family physician visits**						
Starting rate[c]	13.78	13.63	12.29	11.79	9.09	8.73
Week of minimum rate[d]		15MAR2020		15MAR2020		05APR2020
Minimum rate	14.09	11.00	12.46	9.40	9.66	6.07
Week of maximum rate[d]		13SEP2020		13SEP2020		13SEP2020
Maximum rate	14.13	16.10	12.73	14.187	9.49	9.59
Latest rate[e]	14.13	15.50	12.54	13.58	9.46	9.49
**Total specialist visits**						
Starting rate[c]	8.77	8.42	12.36	11.69	7.11	7.02
Week of minimum rate[d]		15MAR2020		15MAR2020		15MAR2020
Minimum rate	8.38	4.66	10.97	6.07	6.74	3.62
Week of maximum rate[d]		13SEP2020		20SEP2020		13SEP2020
Maximum rate	9.12	9.12	11.47	12.82	7.48	7.37
Latest rate[e]	8.84	9.08	11.47	12.82	7.52	7.24
**Total neurologist visits**						
Starting rate[c]	0.56	0.53	3.5	3.3	0.12	0.12
Week of minimum rate[d]		15MAR2020		15MAR2020		15MAR2020
Minimum rate	0.50	0.27	2.92	1.51	0.10	0.06
Week of maximum rate[d]		20SEP2020		16AUG2020		20SEP2020
Maximum rate	0.46	0.61	3.00	4.02	0.11	0.13
Latest rate[e]	0.46	0.61	2.48	3.76	0.11	0.13
**Home care visits**						
Starting rate[c]	206.52	214.25	152.02	157.13	17.01	18.52
Week of minimum rate[d]		26APR2020		05APR2020		05APR2020
Minimum rate	207.85	176.52	153.74	122.92	17.30	15.00
Week of maximum rate[d]		01MAR2020		01MAR2020		01MAR2020
Maximum rate	206.52	214.24	152.02	157.13	17.01	18.52
Latest rate[e]	210.61	212.19	153.31	153.05	17.77	17.87
**Long-term care admission**						
Starting rate[c]	0.27	0.25	0.11	0.10	0.01	0.01
Week of minimum rate[d]		19APR2020		26APR2020		10MAY2020
Minimum rate	0.22	0.02	0.12	0.003	0.006	0.0007
Week of maximum rate[d]		01MAR2020		22MAR2020		08MAR2020
Maximum rate	0.27	0.25	0.11	0.12	0.006	0.007
Latest rate[e]	0.21	0.16	0.08	0.09	0.006	0.003
**Mortality**						
Starting rate[c]	0.17	0.16	0.09	0.08	0.04	0.04
Week of minimum rate[d]		07JUN2020		03MAY2020		02AUG2020
Minimum rate	0.16	0.15	0.11	0.07	0.03	0.03
Week of maximum rate[d]		10MAY2020		31MAY2020		05APR2020

© 2022 Bronskill SE et al. *JAMA Health Forum.*

Maximum rate	0.15	0.21	0.09	0.15	0.04	0.04
Latest rate[e]	0.17	0.17	0.09	0.13	0.03	0.04

[a] Older adults include persons aged 65 years and older without a diagnosis of neurodegenerative diseases.

[b] 2019 - historical period starts the week of March 3rd 2019; 2020 - COVID-19 pandemic period starts the week of March 1st 2020.

[c] The starting rate indicates the weekly rate as of the start of the COVID-19 pandemic period (week starting March 1st 2020) and the equivalent week in 2019 (week starting March 3rd 2019) for all services.

[d] Minimum and maximum rates are calculated based on the minimum and maximum rate during the 2020 pandemic period. Historical 2019 rates are calculated based on rates for the equivalent week a year prior to the 2020 COVID-19 pandemic.

[e] The latest rate indicates the rate as of the latest week during the study period (week of Sept 20th 2020 for the COVID-19 pandemic and week of Sept 22nd 2019 for the historical period) for all services.

© 2022 Bronskill SE et al. *JAMA Health Forum.*

eTable 3. Rate ratios for mortality in community-dwelling persons with dementia, persons with Parkinson disease and older adults[a] comparing the COVID-19 pandemic period with the historical period in Ontario, Canada, by age group

	Rate ratios (95% confidence intervals)		Cumulative change in number of outcomes[c], N
	At highest rate in 2020	At latest week[b]	
**DEMENTIA**			
<75 years	**2.57 (1.41, 4.67)**	1.21 (0.73, 2.02)	134
75-84 years	**1.65 (1.16, 2.34)**	0.89 (0.62, 1.26)	328
≥85 years	**1.41 (1.11, 1.78)**	1.08 (0.85, 1.37)	630
**PARKINSON DISEASE**			
<75 years	2.78 (0.89, 8.73)	2.78 (0.89, 8.73)	37
75-84 years	1.53 (0.78, 2.99)	0.99 (0.44, 2.20)	60
≥85 years	1.73 (0.85, 3.51)	1.44 (0.67, 3.10)	53
**OLDER ADULTS**			
<75 years	1.06 (0.90, 1.25)	1.14 (0.96, 1.35)	413
75-84 years	1.06 (0.91, 1.23)	1.09 (0.93, 1.29)	500
≥85 years	1.14 (0.98, 1.34)	1.05 (0.89, 1.24)	568

Bolded numbers indicate statistical significance at $P < 0.05$.

[a]Older adults include persons aged 65 years and older without a diagnosis of neurodegenerative diseases.

[b]The latest weekly rate is as of September 20, 2020 and September 22, 2019 for all services.

[c]The cumulative change in the number of outcomes was calculated comparing the number of events within the pandemic period (March 1 to September 20, 2020) and historical period (March 3 and September 22, 2019). Negative values indicate a decreased number of events in the 2020 COVID-19 pandemic period.

© 2022 Bronskill SE et al. *JAMA Health Forum.*
